# Regulating NETosis: Increasing pH Promotes NADPH Oxidase-Dependent NETosis

**DOI:** 10.3389/fmed.2018.00019

**Published:** 2018-02-13

**Authors:** Meraj A. Khan, Lijy M. Philip, Guillaume Cheung, Shawn Vadakepeedika, Hartmut Grasemann, Neil Sweezey, Nades Palaniyar

**Affiliations:** ^1^Program in Translational Medicine, Peter Gilgan Centre for Research and Learning, The Hospital for Sick Children, Toronto, ON, Canada; ^2^Department of Laboratory Medicine and Pathobiology, Faculty of Medicine, University of Toronto, Toronto, ON, Canada; ^3^Department of Paediatrics, Faculty of Medicine, University of Toronto, Toronto, ON, Canada; ^4^Institute of Medical Sciences, Faculty of Medicine, University of Toronto, Toronto, ON, Canada; ^5^Department of Physiology, Faculty of Medicine, University of Toronto, Toronto, ON, Canada; ^6^Massachusetts General Hospital, Shriners Hospitals for Children in Boston, Harvard Medical School, Boston, MA, United States

**Keywords:** neutrophil extracellular trap formation, pH, spontaneous NETosis, NADPH-dependent NETosis, lipopolysaccharide-induced NETosis, Gram-negative bacteria-induced NETosis, Gram-positive bacteria-induced NETosis, correcting pH

## Abstract

Neutrophils migrating from the blood (pH 7.35–7.45) into the surrounding tissues encounter changes in extracellular pH (pH_e_) conditions. Upon activation of NADPH oxidase 2 (Nox), neutrophils generate large amounts of H^+^ ions reducing the intracellular pH (pH_i_). Nevertheless, how extracellular pH regulates neutrophil extracellular trap (NET) formation (NETosis) is not clearly established. We hypothesized that increasing pH increases Nox-mediated production of reactive oxygen species (ROS) and neutrophil protease activity, stimulating NETosis. Here, we found that raising pH_e_ (ranging from 6.6 to 7.8; every 0.2 units) increased pH_i_ of both activated and resting neutrophils within 10–20 min (Seminaphtharhodafluor dual fluorescence measurements). Since Nox activity generates H^+^ ions, pH_i_ is lower in neutrophils that are activated compared to resting. We also found that higher pH stimulated Nox-dependent ROS production (R123 generation; flow cytometry, plate reader assay, and imaging) during spontaneous and phorbol myristate acetate-induced NETosis (Sytox Green assays, immunoconfocal microscopy, and quantifying NETs). In neutrophils that are activated and not resting, higher pH stimulated histone H4 cleavage (Western blots) and NETosis. Raising pH increased *Escherichia coli* lipopolysaccharide-, *Pseudomonas aeruginosa* (Gram-negative)-, and *Staphylococcus aureus* (Gram-positive)-induced NETosis. Thus, higher pH_e_ promoted Nox-dependent ROS production, protease activity, and NETosis; lower pH has the opposite effect. These studies provided mechanistic steps of pH_e_-mediated regulation of Nox-dependent NETosis. Raising pH either by sodium bicarbonate or Tris base (clinically known as Tris hydroxymethyl aminomethane, tromethamine, or THAM) increases NETosis. Each Tris molecule can bind 3H^+^ ions, whereas each bicarbonate HCO3^−^ ion binds 1H^+^ ion. Therefore, the amount of Tris solution required to cause the same increase in pH level is less than that of equimolar bicarbonate solution. For that reason, regulating NETosis by pH with specific buffers such as THAM could be more effective than bicarbonate in managing NET-related diseases.

## Introduction

Neutrophils infiltrating tissues during an inflammatory response encounter a range of levels of pH (1–3). Nevertheless, how extracellular pH (pH_e_) regulates NETosis is not clearly understood. In response to different agonists, neutrophils undergo two major types of NETosis (4–6). It has been well-established that bacterial endotoxin, Gram-negative bacteria (e.g., *Pseudomonas aeruginosa*), and Gram-positive bacteria (e.g., *Staphylococcus aureus*) induce NADPH oxidase 2 (Nox) and subsequent ROS production (7–9). We have recently shown that neutrophils undergo increased Nox-dependent NETosis in response to higher doses of lipopolysaccharide (LPS) and increasing microbial load (7). By contrast, increasing intracellular calcium concentration induces citrullination of histones and thereby facilitates Nox-independent NETosis (5, 9). In this study, we address the mechanism by which pH regulates Nox-dependent NETosis.

Paradoxically, neutrophils carry two sets of NETosis-related enzymes, with either acidic or basic pH optima. The pH optima for neutrophil proteases (e.g., elastase, proteinase 3, and cathepsins) and myeloperoxidase (MPO) are basic (pH 7.5–8.5) and acidic (pH 4.7–6.0), respectively (10–12). A recent study showed that acidic pH lowers neutrophil extracellular trap (NET) formation induced by phorbol myristate acetate (PMA) and immune complexes (13). However, the effect of pH on LPS, Gram-positive, and Gram-negative bacteria are unknown. Therefore, we here studied the effect of pH on Nox-dependent NETosis induced by agonists such as PMA, LPS, *P. aeruginosa*, and *S. aureus*. We show that altered pH_e_ rapidly affects neutrophilic intracellular pH (pH_i_), Nox-mediated reactive oxygen species (ROS) production, protease-mediated histone cleavage, and subsequent NETosis. Raising pH through a range from 6.6 to 7.8 increases the level of NETosis. These findings help explain how pH regulates NETosis and demonstrate that pH adjustment could potentially be used to regulate NETosis of migrating neutrophils. Tris is a more effective pH regulator than bicarbonate, therefore, Tris-based buffers could be better for correcting pH and regulating NET-related diseases.

## Materials and Methods

### Research Ethics Board Approval

This study protocol for using human blood samples was approved by the ethics committee of The Hospital for Sick Children, Toronto. All the procedures including healthy human volunteer recruitment for blood donation were performed in accordance with the ethics committee guidelines. All the volunteers participating in this study gave their signed consent prior to blood draw.

### Buffer and Reagent Preparation

SYTOX^®^ Green Nucleic Acid Stain dye, DHR123, and pH_i_ indicator Seminaphtharhodafluor (SNARF) were obtained from Molecular Probes (Thermo Fisher Scientific, Waltham, MA, USA). All buffers, agonists, inhibitors, and other reagents were purchased from Sigma-Aldrich unless otherwise stated. The standard medium of these experiments was RPMI 1640 medium (Invitrogen) supplemented with 10 mM HEPES buffer. The amount of HCl and NaOH was predetermined to change the pH of RPMI media to 6.6, 6.8, 7.0, 7.2, 7.4, 7.6, or 7.8, and these isotonic RPMI media with different pHs were added to the neutrophil suspension for further experiments.

### Primary Human Neutrophils Isolation

The primary human neutrophils were isolated by using a slight modification of the PolymorphPrep (Axis-Shield) protocol as previously reported (4–7). Briefly, the peripheral blood from the healthy male donors was collected in K2 EDTA blood collection tubes (Becton, Dickinson and Co.). Equal volumes of blood were layered over the PolymorphPrep (Axis-Shield) and spun for 35 min at 600 × *g*, 25°C to separate the neutrophil band. After washing the neutrophil band, red blood cells were lysed with a 0.2% (w/v) NaCl hypotonic solution for 30 s followed by an addition of an equal volume of 1.6% (w/v) NaCl solution with 20 mM HEPES buffer to provide the isotonic condition. Isolated neutrophils were resuspended in RPMI medium (Invitrogen) containing 10 mM HEPES (pH 7.2). Neutrophil counting and viability check were done by trypan blue and using a hemocytometer. Furthermore, the purity of neutrophils was determined by Cytospin preparation and imaging. Only neutrophil preparations with >95–98% live and pure were used in the experiments. For each experiment, multiple donors were used to get enough replicates; see details in specific figure legends.

### SNARF Preparation pH_i_ Detection

Isolated neutrophils were treated with 10 μM SNARF^®^-4F 5-(and-6)-Carboxylic Acid (Thermo Fisher Scientific) pH indicator dye and incubated at 37°C for 15 min. After incubation, the cells were washed and suspended in fresh RPMI medium at 1 × 10^6^/mL cells. A volume of 50 μL, containing 50,000 neutrophils was seeded into a 96-well clear bottom black plate (BD Biosciences). Equal volumes (50 μL) of the isotonic RPMI with predetermined pH (6.6, 6.8, 7.0, 7.2, 7.4, 7.6, and 7.8) were added into the wells to adjust the pH of the corresponding wells. Neutrophils buffered with different pHs were stimulated either with only media (−ve control) or PMA. The dual emission spectra of SNARF were measured after adding media (−ve control) and PMA and considered as the zero time point reading. Further pH changes were recorded for every 10 min up to 60 min with an Omega fluorescence microplate reader. The emission spectrum of SNARF undergoes a pH-dependent wavelength shift, and therefore, the ratios (580/640 nm) of the fluorescence intensities from the dye at two emission wavelengths were used for pH_i_ determinations. Carboxy SNARF-4F is typically used by exciting the dye at one wavelength (between 488 and 530 nm) while monitoring the fluorescence emission at two wavelengths, typically at 580 and 640 nm. The fluorescence response of the SNARF indicators has been calibrated with pH-controlled RPMI (6.6, 6.8, 7.0, 7.2, 7.4, 7.6, and 7.8) buffered in the presence and absence of the cells. Each condition was tested with a technical duplicate. Biological replicates (*n*-values; donors) of independent experiments were reported in figure legends.

### ROS Detection Assay

DHR123 dye (Scientific Inc., MA, USA) was used to measure the intracellular ROS production. The neutrophils were preloaded with DHR123 (20 μM) for 10 min as per manufacturer’s instructions with brief modification as we reported earlier (5, 7, 14). After washing the extracellular DHR123 dye, cells were resuspended in fresh RPMI (10 mM HEPES) media and 50 μL of 50,000 cells, seeded into 96-well plates. Furthermore, DHR123-preloaded neutrophils media were adjusted for pHs (6.6, 6.8, 7.0, 7.2, 7.4, 7.6, and 7.8) by adding the equal volume (50 μL) of isotonic RPMI calibrated for respective pHs. These cells were activated with either only media (negative control), PMA, or LPS for another 30 min. The fluorescence was measured every 10 min by an Omega fluorescence microplate reader (900 data points per well) to assess the kinetics of the ROS generation in different conditions. Each condition was tested with a technical duplicate. Biological replicates (*n*-values; donors) of independent experiments were reported in figure legends. Confocal images were acquired as described earlier by counter staining of the nuclei with DAPI in different pH conditions.

### Flow Cytometry

Flow cytometry was used for validating the ROS production in neutrophils at different pH_e_ conditions. Cells were treated with 20 μM DHR123 for 10 min at 37°C. These preloaded cells were washed and resuspended in fresh media, and their pHs were adjusted to 6.6, 7.4, and 7.8. These cells with adjusted pH_e_s were activated with either media only (−ve control) or PMA for 30 min, and the ROS production was analyzed by counting 10^4^ events using the 488 filter and the FITC laser of the Gallios (Beckman Coulter Inc.) flow cytometer. Data were analyzed by using Flow Jo software.

### Sytox Green NETosis Assay

A cell impermeable DNA binding Sytox Green dye (Life Technologies) was used for measuring the real-time kinetics of NET release under different conditions. A volume of 50 μL media containing 50,000 neutrophils mixed with 5 μM Sytox green were seeded into 96-well plates. Equal volumes of the isotonic RPMI media with double the strength of different pHs were added into the respective wells to make the final pH 6.6, 6.8, 7.0, 7.2, 7.4, 7.6, and 7.8 of the corresponding wells. These neutrophils under different pHs were activated with agonists, only media (−ve control), PMA, LPS, *P. aeruginosa*, and *S. aureus*. The changes in green fluorescence signal was measured every 30 min for up to 240 min using a fluorescence plate reader (504 nm excitation and 523 nm emission, POLARstar OMEGA, BMG Labtech). By using this POLARstar OMEGA plate reader, 900 data points per well were scanned at each time point to quantify the fluorescence intensity. Total DNA (100% DNA) present in these neutrophils was determined by the fluorescence values of the cells lysed with 0.5% (v/v) Triton-X-100. To calculate percentage of NETosis in each condition, the green fluorescence at time 0 min was subtracted from the fluorescence at each time point and was then divided by the fluorescence values of cell lysed with 0.5% (v/v) Triton X-100. All the experimental values were standardized to total DNA at each time point. Each condition was tested with a technical duplicate. Biological replicates (*n*-values; donors) of independent experiments were reported in figure legends.

### Immunofluorescence Confocal Imaging

A volume of 100 μL RPMI medium containing 100,000 neutrophils were seeded into 12-well chamber slides (BD Falcon). Chamber slides were used for obtaining the high-resolution images. The pH of the respective chamber was adjusted as explained earlier by adding an equal volume of the isotonic RPMI with different pHs (6.6, 7.4, and 7.8). The neutrophils were activated with either media only (−ve control), PMA, LPS, *P. aeruginosa*, or *S. aureus* for 120 min. The cells and NETs were fixed with paraformaldehyde [4% (w/v) for 10 min; 2% (w/v) for overnight] and immunostained with various NET markers. Mouse anti-MPO antibody (ab25989; Abcam) at 1:500 dilution was used for staining MPO (with secondary antibody conjugated with a green fluorescence Alexa fluor 488 dye; 1:2,000 dilution; Thermo Fisher Scientific), while rabbit anti-citrullinated histone 3 antibody (ab5103; Abcam, Lot # GR273046-3) at 1:500 dilution was used for detecting the presence of citrullinated histone H3 (CitH3, with secondary antibody conjugated with a far-red fluorescence dye Alexa fluor 647; 1:1,000 dilution; Thermo Fisher Scientific). DNA was stained with DAPI (1:1,000 dilution). After treating the secondary antibody, slides were washed and mounted by glass cover slips (Fisher Scientific) with anti-fade fluorescent mounting medium (Dako). The images were then taken using an Olympus IX81 inverted fluorescence microscope with a Hamamatsu C9100-13 back-thinned EM-CCD camera and Yokogawa CSU × 1 spinning disk confocal scan head with Spectral Aurora Borealis upgrade, four separate diode-pumped solid-state laser lines (Spectral Applied Research, 405, 491, 561, and 642 nm). The images were taken at 40×/0.95 magnification and processed by Volocity software (version 6.3, Cell Imaging Perkin-Elmer). Immunostained confocal images were quantified by counting normal neutrophils (multilobed nuclei), decondensed (nuclei lost multilobed shape), cloud-like puffed extracellular DNA, and extended extracellular DNA (NET-like structure) to determine the degree of NETosis.

### Immunoblot Analysis

For the immunoblot analysis, the tubes containing 1 × 10^6^ cells with adjusted pHs (6.6, 7.0, 7.4, and 7.8) in each experimental condition were activated either by negative control (only media) or by PMA for 120 min. After incubation, the tubes were placed on ice for 10 min and then centrifuged at 20,000 rcf at 4°C for 10 min. The supernatant was then discarded, and the cell pellets were lysed using the lysis buffer containing 1% (w/v) Triton X-100, 25 mM NaF, 50 mM Tris, 10 mM KCl, 10 μg/mL aprotinin, 2 mM PMSF, 1 mM levamisole, 1 mM NaVO_3_, 0.5 μM EDTA, 25 μM leupeptin, 25 μM pepstatin, one protease inhibitor cocktail tablets per 5 mL (Roche), and one phosphatase inhibitor cocktail tablet per 10 mL (Roche). The samples were then vortexed for 10 s followed by three times of sonication using an aquasonic sonicator (VWR, model 50D at the highest power setting), at 8–10°C, 3 min each. A quarter volume of 5 × loading dye [125 mM Tris HCl at pH 6.8, 6% (w/v) SDS, 8% (v/v) β-mercaptoethanol, 18% (v/v) glycerol, 5 mM EDTA, 5 mM EGTA, 10 μg/mL leupeptin, 10 μg/mL pepstatin, 10 μg/mL aprotinin, 10 mM NaF, 5 mM NaVO3, and 1 mM levamisole] was added followed by 10 min of heating at 95°C with 350 rcm shaking. The samples were separated in a 5% (w/v) stacking and 10% (w/v) resolving gel at 100 V and transferred on a nitrocellulose membrane for 90 min at 400 mA. After transfer, the membranes were blocked with 5% (w/v) milk or BSA in 0.05% phosphate-buffered saline (PBS) with 0.1% Tween (PBST) for 1 h at room temperature. The membranes were incubated with the primary antibody at 4°C overnight followed by three washes with PBST for 30 min. The antibodies used were as follows: anti-Histone H4 (ab16483; Abcam) rabbit pAb at 1:1,000 and anti-GAPDH (FL-335; Santa Cruz) rabbit pAb at 1:2,500. The membranes were then incubated in the secondary antibody solution for 1 h and then washed three times with 0.1% PBST for 30 min. The secondary antibodies used were as follows: donkey anti-rabbit IgG-HRP (31458; Thermo Fisher) at 1:7,500. The densitometry analysis of the blots was done using the Image Studio software (LI-COR Biotechnology) and normalized to the GAPDH.

### Bacterial Culture

*Pseudomonas aeruginosa* and *S. aureus* were selected from single colonies in LB-agar plates and grown overnight in sterile LB broth. Overnight bacterial growth culture was diluted by a factor of 10 and subcultured for 3 h to eliminate the dead colonies or bacteria. Bacterial culture was harvested and washed three times in 5 mL of PBS (pH 7.4) by centrifugation at 5,000 × *g* for 5 min at 4°C. The bacterial concentration was determined by taking optical density (OD) at 600 nm. Furthermore, the multiplicity of infection was calculated by using the colony-forming unit (CFU) formula established in the laboratory by empirical methods: (CFU) × 10^8^ = (OD_600_) × 30.88 − 99.61.

### Statistical Analysis

Statistical analysis was performed using GraphPad Prism statistical analysis software (Version 5.0a). Student’s *t*-test was used for comparing two groups, and for more than two groups, ANOVA with Bonferroni’s posttest or Dunnett’s test was used where appropriate. The technical repeats and applied statistics are mentioned in each Figure legends. A *p*-value of ≤0.05 was considered to be statistically significant. All data are presented as mean ± SEM.

## Results

### High pH Promotes Spontaneous and PMA-Induced NETosis

**Figure 1:**
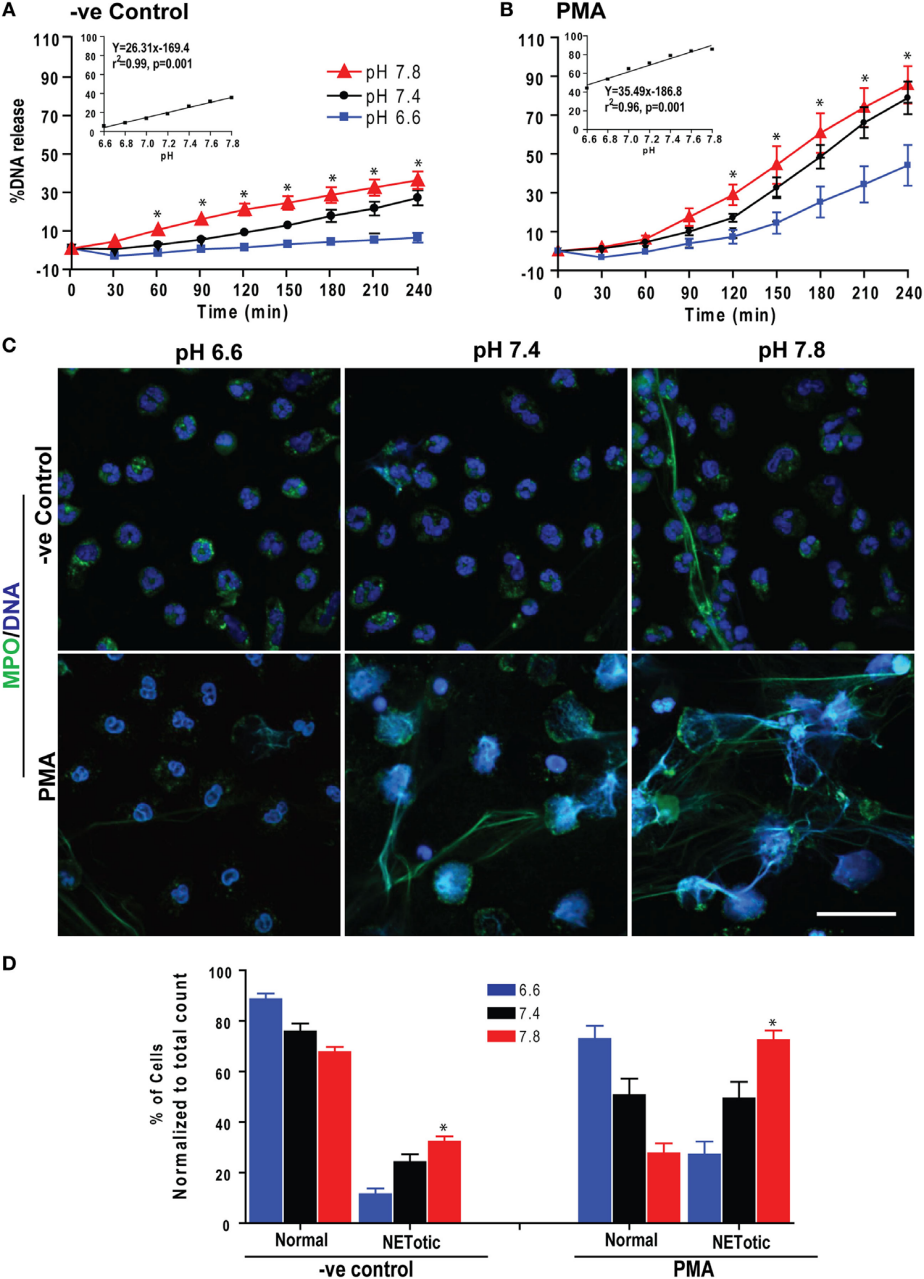
Low extracellular pH (pH_e_) suppressed spontaneous and phorbol myristate acetate (PMA)-mediated NETosis. Neutrophils resuspended in media of predetermined pH_e_ (6.6, 6.8, 7.0, 7.2, 7.4, 7.6, and 7.8) containing 5 μM Sytox Green dye were activated by either medium (−ve control) or PMA. Florescence was recorded by a plate reader for every 30 min up to 4 h. The percentage of DNA release (NETosis) was reported in comparison to an assigned value of 100% for the total DNA released by neutrophils lysed by triton-X. **(A)** The percentage of DNA release kinetics: neutrophils in higher pH_e_ showed more NETosis. The inset regression graph of all seven pH conditions at 240-min time points showed a linear increment of the NETosis with increasing pH. **(B)** In neutrophils activated with PMA, low pH suppressed the PMA-induced NETosis parallel to decreased pH gradient, as shown in the kinetics and respective inset linear regression plots (*n* = 4–5; *p < 0.05; two-way ANOVA with Bonferroni’s posttest conducted at each time point; best fit linear regression analysis; error bars represent SEM). **(C)** Neutrophils resuspended in media of pH 6.6, 7.4, and 7.8 were activated by medium (−ve control) or PMA for 120 min. The fixed cells were immunostained for myeloperoxidase (MPO) and DNA. Confocal images showed the intact morphology of the nuclei in media control and PMA-activated neutrophils in low pH media. At higher pH, MPO colocalized to neutrophil extracellular trap (NET)-DNA generated by control and PMA, although the amount of NETosis was much higher in PMA-activated compared to control cells (blue, DAPI staining for DNA; green, MPO; *n* = 3; scale bar 20 μm). **(D)** The percentages of normal, decondensed, or puffed and NET-like (NETotic) cells were calculated based on the MPO, DAPI staining, MPO-DAPI colocalization, and nuclear morphology. The quantitative analyses confirm the qualitative analyses of the NETs status in neutrophils under pH conditions either treated by media only (−ve control) or PMA (*n* = 3; *p < 0.05, comparing the condition between pH 6.6 and 7.8; one-way ANOVA with Tukey’s multiple comparison posttest). See additional details in Figure S1 in Supplementary Material.

We first determined the effect of pH on spontaneous and Nox-dependent NETosis. We incubated purified peripheral blood neutrophils (PMNs) at pH above or below physiological blood pH (6.6–7.8; in 0.2 pH increments) with or without the prototypic agonist PMA to induce Nox-dependent NETosis. Monitoring Sytox green fluorescence (a proxy for extracellular NET DNA) every 30 min for 4 h showed that raising pH increased the rate and amount of NETosis (Figures 1A,B). To confirm true NETosis, we conducted confocal immunofluorescence microscopy. These images showed colocalization of MPO and NET DNA and confirmed that resting and PMA-stimulated neutrophils formed greater amounts of NETs with increasing pH (Figures 1C,D; Figure S1 in Supplementary Material). A regression line at the final time point showed a clear increase in Sytox Green readings with increasing pH (Figures 1A,B, inset). The slope of spontaneous NETosis was less steep than that of PMA-mediated NETosis, indicating that the rate of spontaneous NETosis was lower than that of PMA-mediated NETosis. Overall, pH affected both spontaneous and PMA-mediated NETosis; pH above the normal blood pH of ~7.4 promoted NETosis, whereas a more acidic pH suppressed NETosis.

### Changes in pH_e_ Rapidly Altered pH_i_

**Figure 2:**
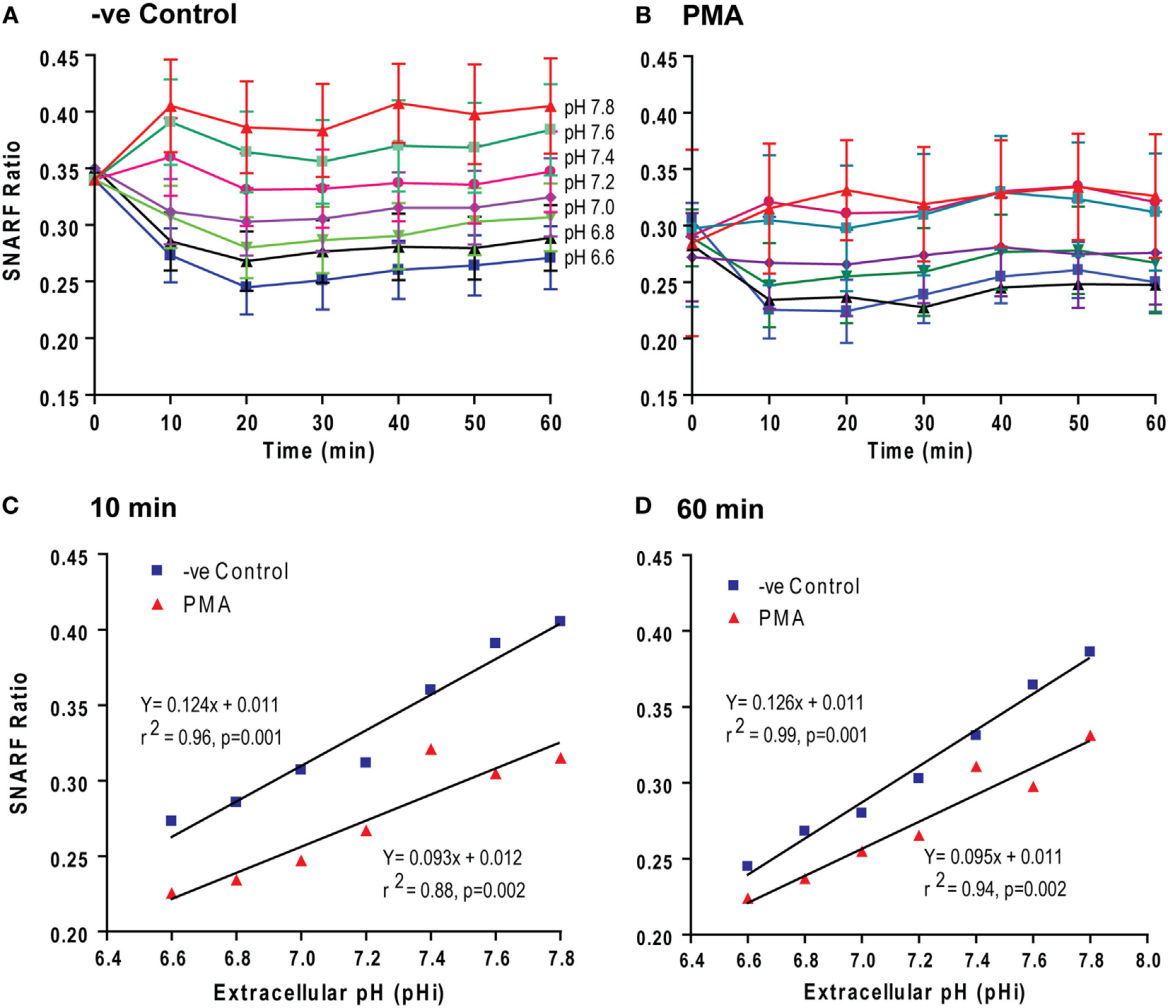
Extracellular pH (pH_e_) altered intracellular pH (pH_i_) within 10–20 min. pH_i_ of the neutrophils activated with media (−ve control) and phorbol myristate acetate (PMA) was estimated from the excitation ratios of Seminaphtharhodafluor (SNARF) fluorescence. **(A,B)** The time course of the fluorescence ratio analysis showed that the change in pH_e_ was followed by a pH_i_ change within 10–20 min. **(C,D)** Linear regression for two time-points (10 and 60 min) showed that the pH_i_ values of PMA-treated neutrophils were lower than the control neutrophils. However, the slopes of the graphs at 10- and 60-min different time points were not different (*n* = 3–4; *p*-value in each graph shows whether the slope is different than 0; error bars represent SEM).

To determine the mechanism that governs pH-mediated NETosis, we next tested how pH_e_ changes affect pH_i_. We used a pH-sensitive dual-wavelength SNARF dye for this purpose. SNARF-loaded neutrophils were suspended in media with six different levels of pH_e_ ranging from 6.6 to 7.8, in the presence or absence of PMA. Changes in pH_i_ were monitored by SNARF wavelength ratios every 10 min up to 1 h. We limited our analysis to 1 h because neutrophils in both control and PMA conditions are viable up to this time point (Figure 1; Sytox assays also indicate cell permeability), avoiding the extracellular buffers entering any dead cells. Within 10–20 min, the changes in pH_i_ of the neutrophils reflected the changes in pH_e_ in both control and PMA conditions (Figures 2A,B). The pH_i_ values of PMA-treated neutrophils were lower than in control neutrophils (the SNARF ratio difference was 0.62; *p* = 0.001 and *p* = 0.002) over the entire pH_e_ range studied; however, the pH_i_ did not change substantially after 10 min (slope of the graphs at 10 and 60 for controls: 0.124 vs. 0.126; for PMA: 0.093 vs. 0.095) (Figures 2C,D). Activated Nox catalyzes the oxidation of NADPH and generates NADP^+^, electron (e^−^), and H^+^, concomitantly transports e^−^ out of the cytoplasm but leaves the NADP^+^ and H^+^ in the cytoplasm; hence, pH_i_ should be lower in PMA-activated neutrophils than control neutrophils. The data obtained in this set of experiments indicate that activated neutrophils had an acidic pH_i_, which was further reduced by low pH_e_.

### Higher pH Stimulated ROS Production in both Resting and PMA-Stimulated Neutrophils

**Figure 3:**
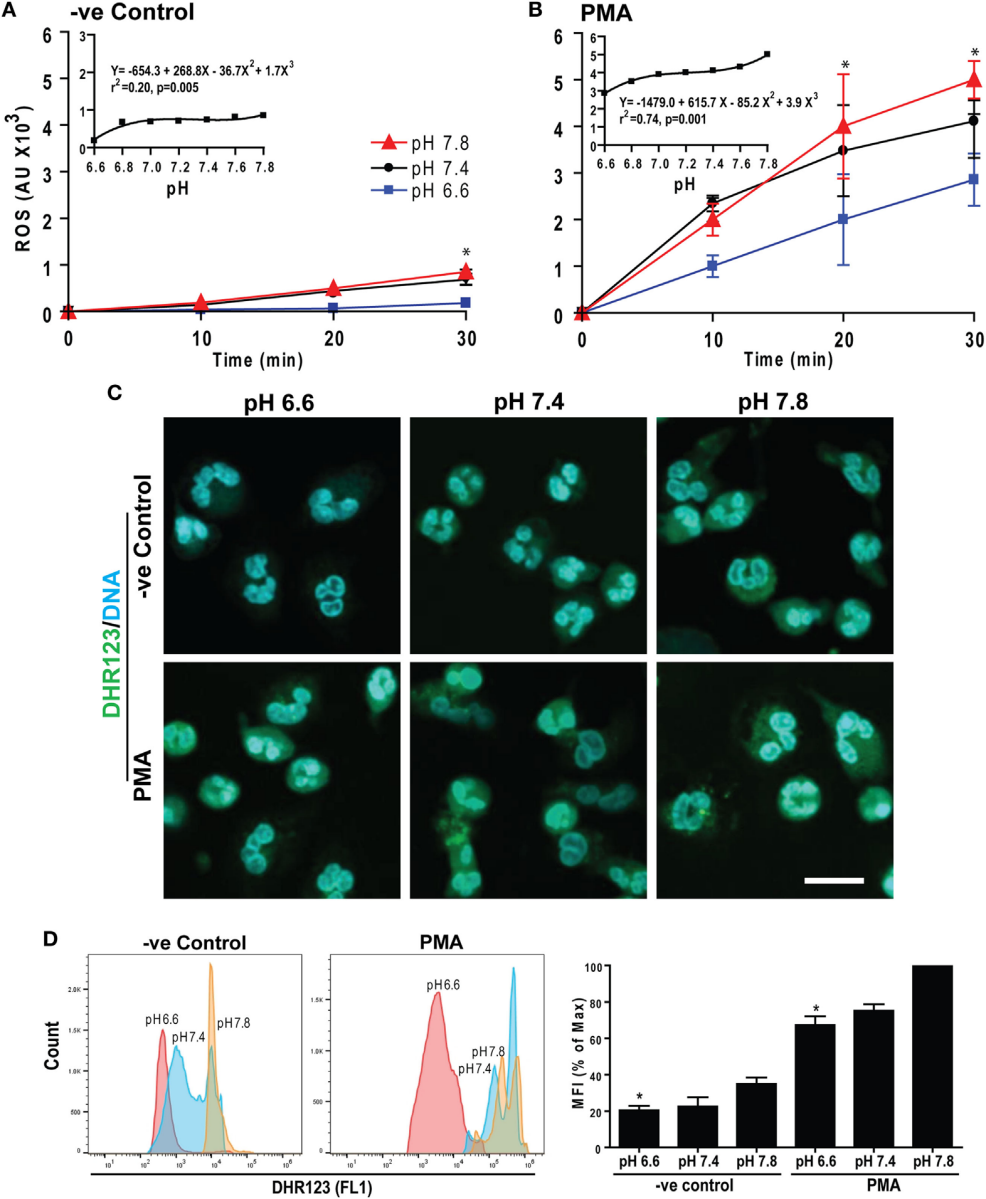
Raising extracellular pH increased reactive oxygen species (ROS) production. Neutrophils preloaded with DHR123 ROS indicator dye in media of various pH values were activated with media (−ve control) or phorbol myristate acetate (PMA). The ROS generation kinetics was estimated by a fluorescence plate reader up to 30 min post activation. **(A,B)** The R123-based ROS generation kinetics showed that elevating pH increased ROS production in both control and PMA-treated neutrophils. As shown in the inset regression plot, the magnitude and the rate of ROS production were higher for PMA-treated neutrophils than control neutrophils (*n* = 3–4; *p < 0.05, between pH 6.6 and 7.8 conditions at respective time points; two-way ANOVA with Bonferroni’s posttest conducted at each time point; best fit non-linear polynomial second-order regression analysis; *p*-value in each inset graph shows whether the slope is different than 0; error bars represent SEM). **(C)** The ROS generation in neutrophils activated with either control or PMA at pH 6.6, 7.4, or 7.8 was imaged by confocal microscopy. The R123 (green) and DNA (blue) fluorescence staining at 30 min showed more ROS at higher pH in both control and PMA-activated neutrophils, although the amount was greater in PMA-treated cells (*n* = 3; scale bar 20 μm). **(D)** Flow cytometry analyses were performed to detect the ROS production in each cell. DHR123-preloaded neutrophils were activated either by media (−ve control) or PMA for 30 min at different pH conditions (pH 6.6, 7.4, and pH 7.8). Mean fluorescence intensities (percentage of maximum) showed higher ROS production at higher pH (*n* = 3; *p < 0.05, between pH 6.6 and 7.8 conditions; one-way ANOVA with Dunnett’s posttest).

The e^−^ generated by Nox reacts with molecular O_2_ to generate superoxide among other ROS, which are important for NETosis (15, 16). Therefore, we measured intracellular ROS by DHR123 as previously described (5, 7, 14). Neutrophils were preloaded with DHR123 and activated with or without PMA at six different pH levels. Generation of oxidized green florescence R123 in these neutrophils was monitored every 10 min for 30 min. Raising pH stimulated ROS production in both control and PMA-treated neutrophils (Figures 3A,B). The magnitude and rates of ROS production were higher for PMA-treated than control neutrophils (see insets). To confirm increased ROS production, we imaged these cells as well as performed flow cytometry analyses at 30 min where ROS production was high in PMA-treated cells. These data sets show that increasing pH increased ROS production. Elevating pH also increased production of ROS in control neutrophils, albeit to a lesser degree (Figures 3C,D). Therefore, raising pH increased ROS production in neutrophils, and the pH effect was enhanced in the presence of the activators of neutrophils.

### Nox Regulated the pH-Dependent Increase in Spontaneous and PMA-Mediated NETosis

**Figure 4:**
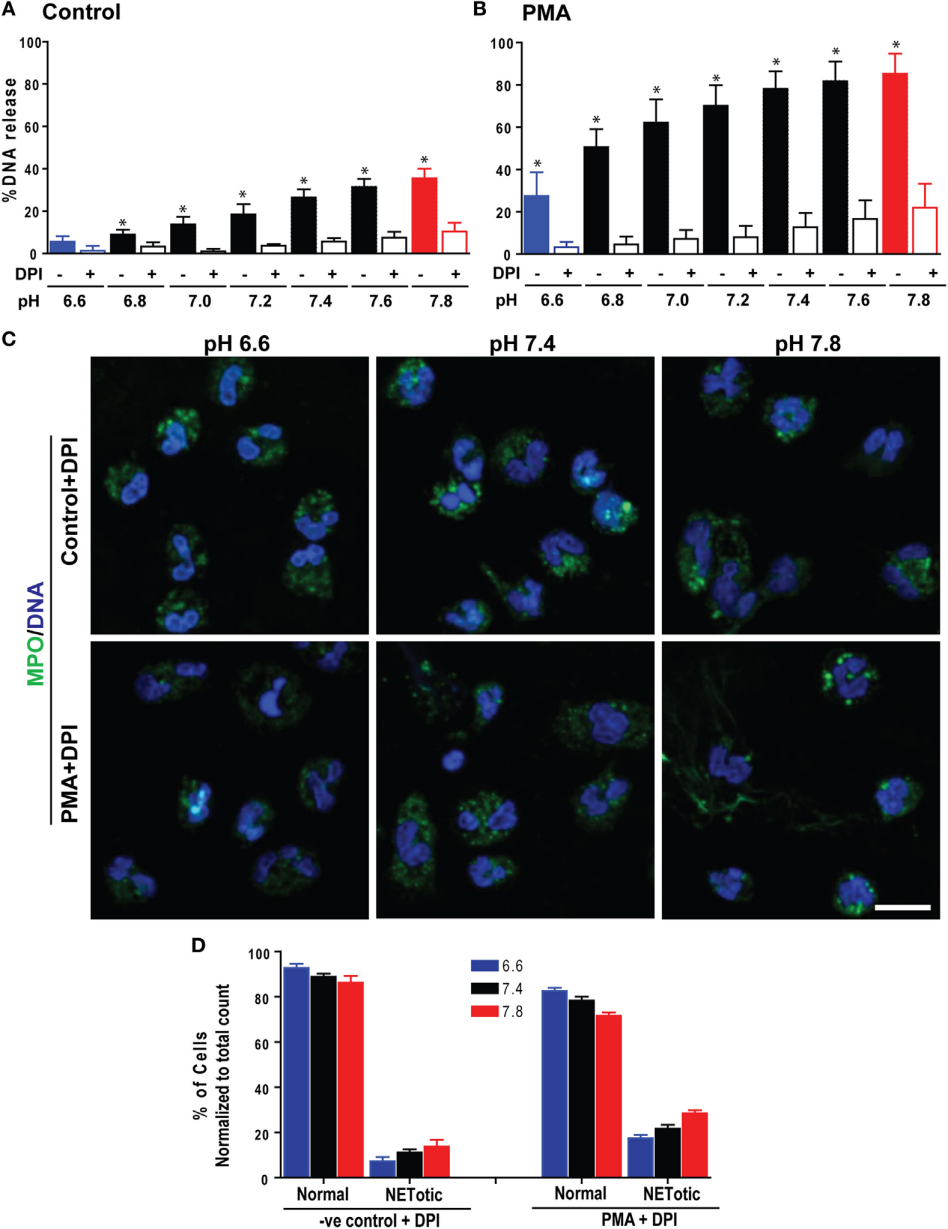
The Nox inhibitor diphenyleneiodonium (DPI) suppressed a pH-dependent increase in spontaneous and phorbol myristate acetate (PMA)-mediated NETosis. The Sytox Green NETosis kinetics were performed using neutrophils at different pHs, activated by medium only (−ve control) or PMA in the presence or absence of the Nox inhibitor, DPI. **(A,B)** As shown in the percentage of DNA release bar graph, DPI suppressed both pH-dependent increases in spontaneous and PMA-mediated NETosis (*n* = 3–4; *p < 0.05; one-way ANOVA with Tukey’s multiple comparison posttest; error bars represent SEM). **(C)** Confocal images of the myeloperoxidase (MPO) and DNA staining confirmed the spontaneous and PMA-mediated NETosis suppression (blue, DAPI staining for DNA; green, MPO; *n* = 3; scale bar 20 μm). **(D)** The percentages of normal, decondensed or puffed, and neutrophil extracellular trap (NET)-like (NETotic) cells were calculated based on the MPO, DAPI staining, MPO-DAPI colocalization, and nuclear morphology. The quantitative analyses confirm the qualitative analyses of the NET status in neutrophils under pH conditions either treated by media only (−ve control) or PMA with DPI (*n* = 3; one-way ANOVA with Tukey’s multiple comparison posttest). See Figure S2 in Supplementary Material for the NETosis kinetics tracing and regression slope of the NETosis suppression.

Neutrophils can generate ROS either by Nox or mitochondria (17, 18). To determine whether pH-dependent NETosis is attributable to Nox-mediated ROS production, we repeated the NETosis assays, in the presence or absence of the Nox inhibitor diphenyleneiodonium (DPI). Sytox Green plate reader assays (Figures 4A,B; Figure S2 in Supplementary Material) and confocal microscopy (Figures 4C,D) indicated that DPI suppresses both spontaneous and PMA-mediated NETosis. Therefore, like PMA-mediated NETosis, spontaneous NETosis was also dependent on Nox activity.

Taken together, these results (Figures 1–4) showed that alterations in pH_e_ led to pH_i_ adjustments within 10–20 min; elevating pH stimulated Nox activity and subsequent ROS production and thereby enhancing spontaneous and PMA-mediated NETosis (PMA > > no PMA). Higher pH facilitated these steps, whereas low pH exerted the opposite effect, suppressing both spontaneous and PMA-mediated NETosis.

### Higher pH Promoted Histone Cleavage and Modification during NETosis

**Figure 5:**
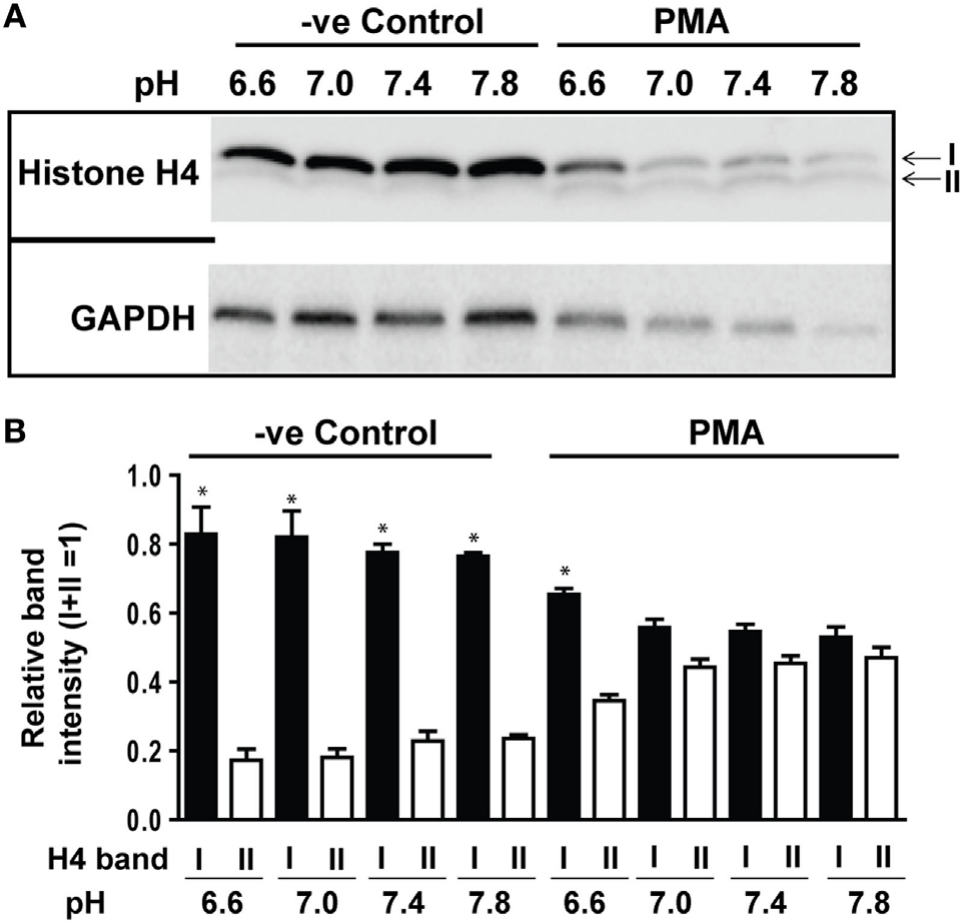
Elevating pH promoted histone cleavage. Histone H4 immunoblot analysis was performed by using neutrophils stimulated with either medium (−ve control) or phorbol myristate acetate (PMA) in different pH conditions (6.6, 7.0, 7.4, and 7.8). **(A)** Histone H4 immunoblot showed a pH-dependent increase in H4 cleavage during PMA-mediated NETosis, without substantial cleavage observed in spontaneous NETosis. GAPDH blots were used as loading controls (*n* = 3). **(B)** The densitometry data of each H4 band were normalized to the total intensities of both bands. The densitometry data showed the increased pH gradient promotes H4 cleavage in PMA-mediated NETosis (*n* = 3; *p < 0.05, comparing between bands I and II at their respective pH conditions; one-sample *t*-test compared to each band). See Figure S4 in Supplementary Material, for the full Western blot images.

In addition to ROS generation, cleavage of histones by granular proteases is a key step in Nox-dependent NETosis. Therefore, we determined H4 cleavage by Western blot analysis during spontaneous and PMA-mediated NETosis across a range of pH conditions. No substantial differences were detected during spontaneous NETosis. By contrast, a clear pH-dependent increase in H4 cleavage was detected during PMA-mediated NETosis. Standardizing H4 cleavage with GAPDH confirmed the pH-dependent increase in histone cleavage (Figures 5A,B). The intensity of all protein bands decreased with increasing pH, suggesting that neutrophil proteases were more active at higher pH. Therefore, neutrophil proteases that are more active at higher pH promote histone cleavage in PMA-mediated NETosis under more alkaline conditions.

We also examined the degree of CitH3 formation by immunocytochemistry to examine the relevance of PAD4 in the pH-dependent increase in Nox-dependent NETosis. Some degree of CitH3 formation (based on the qualitative examination of CitH3 immunostaining) was detected at higher pH conditions. The few NETotic neutrophils present at higher pH showed CitH3, whereas the intact neutrophils did not (Figure S3 in Supplementary Material). Nevertheless, CitH3 is hardly detectable in PMA-mediated NETosis compared to the positive controls (neutrophils activated with calcium ionophore A23187). As expected, the degree of CitH3 formation was much less in PMA-mediated Nox-dependent NETosis than calcium ionophore-mediated NETosis (4, 5). Therefore, raising pH induced a modest degree of CitH3 formation, but the contribution of CitH3 to Nox-dependent NETosis is low.

### pH Changes Had Similar Effects on LPS- and Bacteria-Induced NETosis

**Figure 6:**
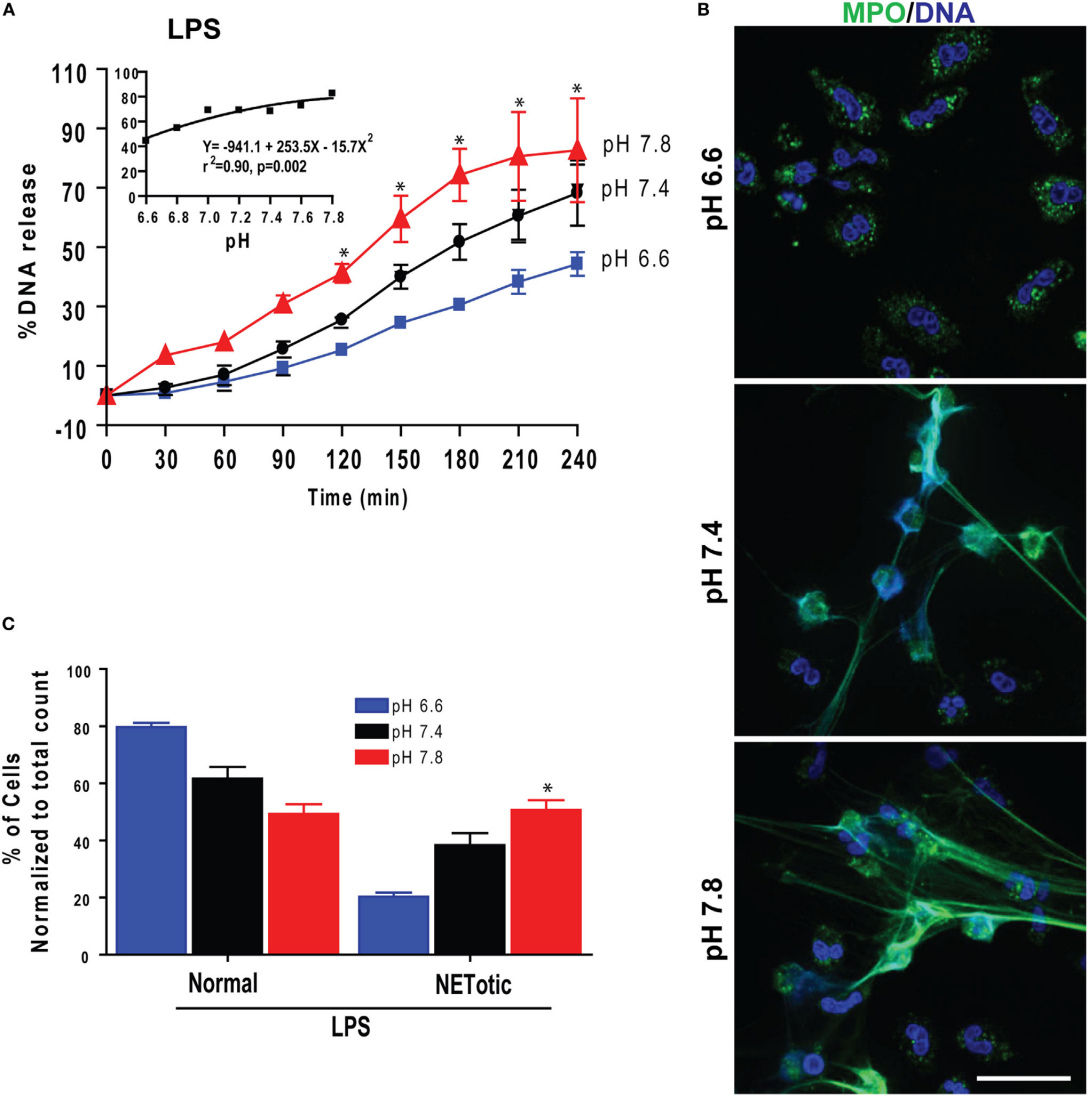
Elevating pH increased lipopolysaccharide (LPS)-mediated NETosis. **(A)** Sytox Green NETosis kinetics was performed in neutrophils activated with LPS (25 μg/mL) under conditions of pH 6.6, 6.8, 7.0, 7.2, 7.4, 7.6, or 7.8. The percentage of DNA release data showed that elevated pH promoted more NETosis in LPS-activated cells. The inset regression graph of the seven pH tracings showed an increment of the NETosis during elevated pH conditions in LPS-activated cells (*n* = 4–5; *p < 0.05, between pH 6.6 and 7.8 conditions at specified time points; two-way ANOVA with Bonferroni’s posttest conducted at each time point; best fit non-linear polynomial second-order regression analysis; error bars represent SEM). **(B)** Confocal images of the myeloperoxidase (MPO) and DNA staining confirmed that the increasing pH increased LPS-mediated NETosis (blue, DAPI staining for DNA; green, MPO; *n* = 3; scale bar 20 μm). **(C)** The percentages of cell counting (quantitative analyses) confirm the qualitative analyses of the neutrophil extracellular trap (NET) status in neutrophils under pH conditions by LPS (*n* = 3; *p < 0.05, between pH 6.6 and 7.8 conditions; one-way ANOVA with Tukey’s multiple comparison posttest).

Gram-negative bacteria, their cell wall component LPS, and Gram-positive bacteria induce Nox-dependent NETosis (5, 7, 19, 20). Therefore, to determine the effect of pH on biologically relevant NETosis-inducing agonists, we first tested the effect of pH on LPS-mediated NETosis. Sytox Green plate reader assays and imaging showed that raising pH stimulated LPS-mediated NETosis (Figure 6A). Regression analyses conducted at the last time point showed a clear pH-dependent effect of NETosis (inset). Immunofluorescence confocal microscopy and quantifying neutrophil with different nuclear morphology and extracellular DNA confirmed the Sytox Green plate reader assay findings (Figures 6B,C). Therefore, as with PMA-mediated Nox-dependent NETosis, elevating pH stimulated LPS-mediated NETosis.

**Figure 7:**
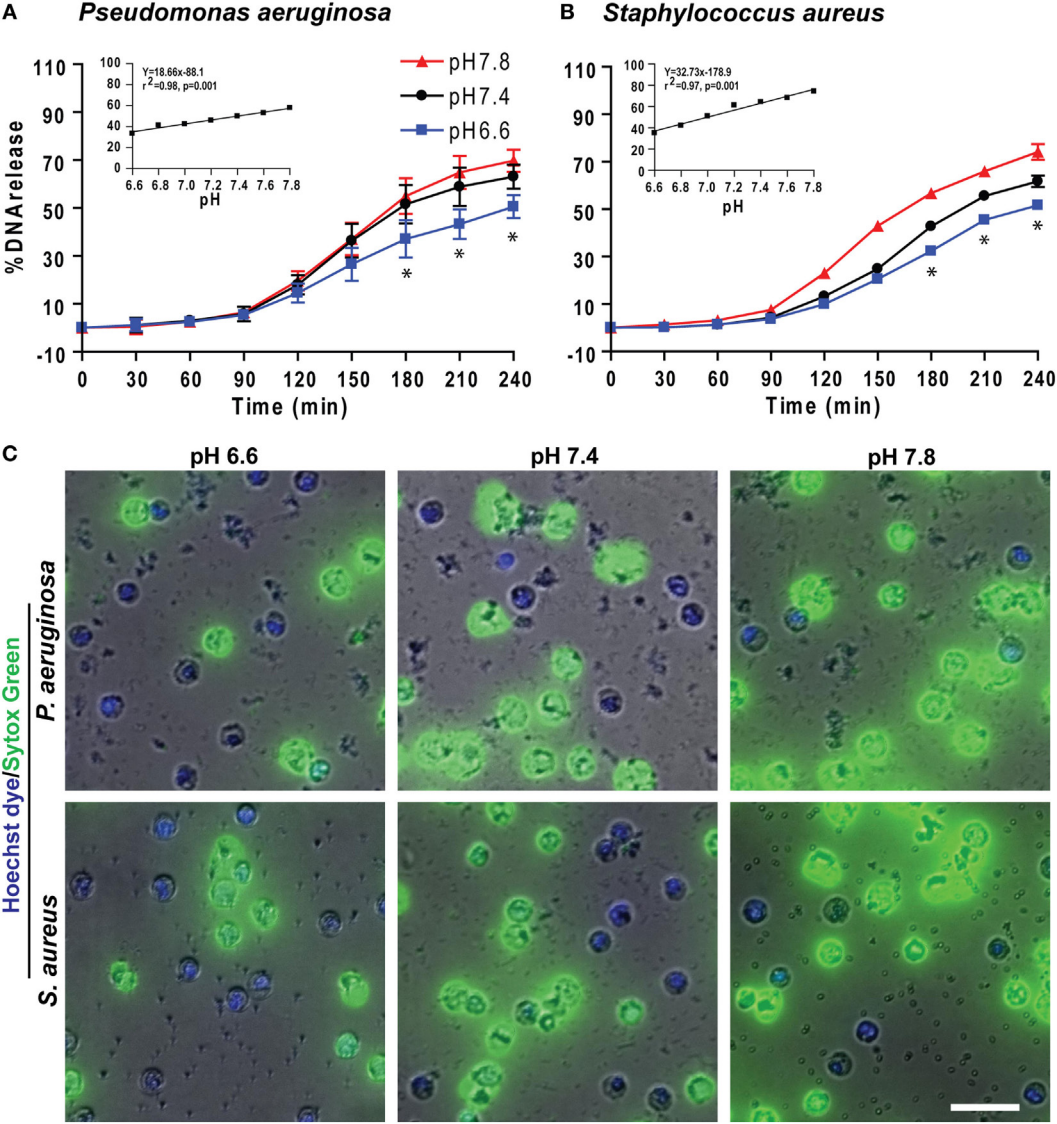
Elevating pH increased *Pseudomonas aeruginosa* and *Staphylococcus aureus* bacteria-mediated NETosis. **(A,B)** A typical tracing of the percentage of DNA release kinetics of the neutrophils activated either by *P. aeruginosa* or *S. aureus* showed more NETosis at higher pHs. The inset regression graph of all seven pH conditions at 240-min time points showed a linear increment of the NETosis with increasing pH (*n* = 3–4; best fit linear regression analysis; *p < 0.05, comparing between pH 6.6 and 7.8 conditions). **(C)** Live cell images of the neutrophils activated with either by *P. aeruginosa* or *S. aureus* was performed at 120-min post activation. Live cell images stained with Hoechst dye and Sytox Green showed more NETosis occurring in response to bacteria at higher pHs (blue, Hoechst dye staining live cell DNA; green, Sytox Green impermeable DNA staining dye; *n* = 3, scale bar 20 μm).

We also tested the effect of pH on NETosis induced by pathogens such as the Gram-negative bacterium *P. aeruginosa* and Gram-positive bacterium *S. aureus*. Like PMA and LPS conditions, more NETosis occurred in response to bacteria under more alkaline conditions (Figure 7). Collectively, these data (Figures 1–7) showed that raising pH increased ROS production, protease activity, and NETosis induced under baseline conditions, as well as by PMA, LPS, Gram-negative, and Gram-positive bacteria. The pH effect was much higher when the NETosis was induced by an agonist.

### Correcting Low pH by Sodium Bicarbonate and Tris Base Corrects NETosis

**Figure 8:**
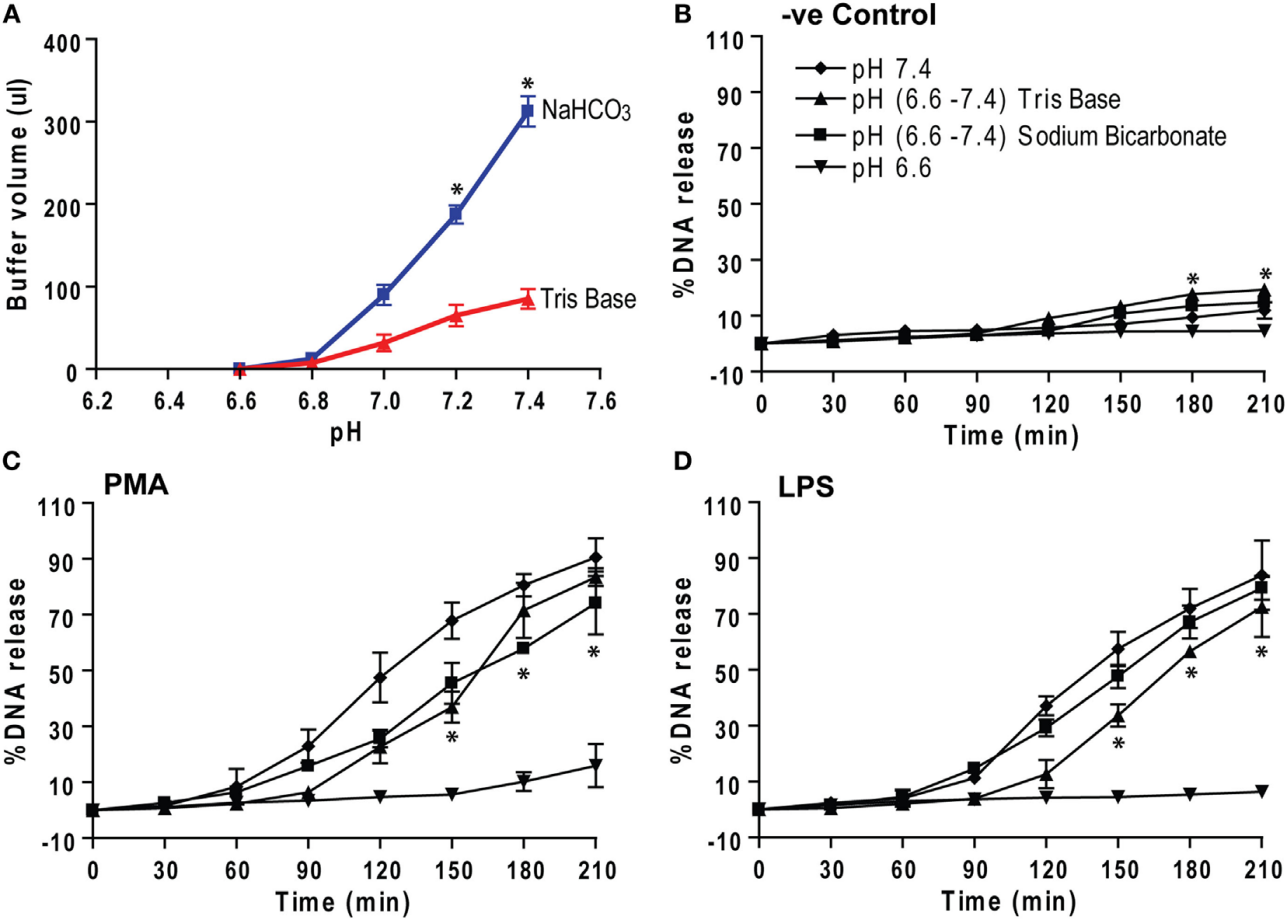
Correcting low pH by sodium bicarbonate and Tris base (THAM) corrects NETosis. **(A)** The low pH media (pH 6.6) were raised by adding either equimolar sodium bicarbonate or Tris base to raise pH to different levels. The titration analyses show that much higher volume of sodium bicarbonate solution is needed to correct the pH than Tris solution. **(B–D)** Neutrophils in low pH (6.6) media containing 5 μM Sytox Green dye were activated by either negative control (media), phorbol myristate acetate (PMA), or lipopolysaccharide (LPS). After 30 min, pH of the buffer was increased either sodium bicarbonate or Tris to 7.4. **(B–D)** The percentage of DNA release kinetics show that pH correction either by sodium bicarbonate or Tris base, clinically known as THAM (from 6.6 to 7.4), increases NETosis compared to low pH (6.6) condition. Collectively, the data show that the pH correction (from 6.6 to 7.4) helps neutrophils to undergo NETosis (*n* = 3; *p < 0.05, comparing between pH 6.6 and Tris base (6.6–7.8) conditions; two-way ANOVA with Bonferroni’s posttest conducted at each time point; one-sample *t*-test). See Figure S5 in Supplementary Material, for the percentage of DNA release bar graph of the last time point (210 min).

Different compounds can be used for adjusting pH. Bicarbonate and Tris are two compounds that can be used to increase pH in humans. Theoretically, each Tris molecule can bind to three H^+^ ions, whereas each bicarbonate ion can neutralize one H^+^ ion. Hence, we tested the efficiency of these compounds in modulating NETosis. To increase the pH of the media from 6.6 to various pHs, Tris solutions required smaller volumes than the equimolar bicarbonate solutions (Figure 8A). Neutrophils in low pH (6.6) media were activated by negative control (medium), PMA, or LPS to induce NETosis. After 30 min, the pH was adjusted by adding precalculated amounts of sodium bicarbonate or Tris to 7.4, and NETosis kinetics were recorded. The percentage of DNA release graphs show that the pH elevation from 6.6 to 7.4 enabled these neutrophils to undergo effective NETosis (Figures 8B–D). Therefore, both bicarbonate and Tris base (or THAM) could be used for increasing the pH and subsequently promoting NETosis. Tris requires much smaller volumes than bicarbonate solutions to adjust the pH, hence, this trivalent molecule may be a better treatment option than bicarbonate.

## Discussion

**Figure 9:**
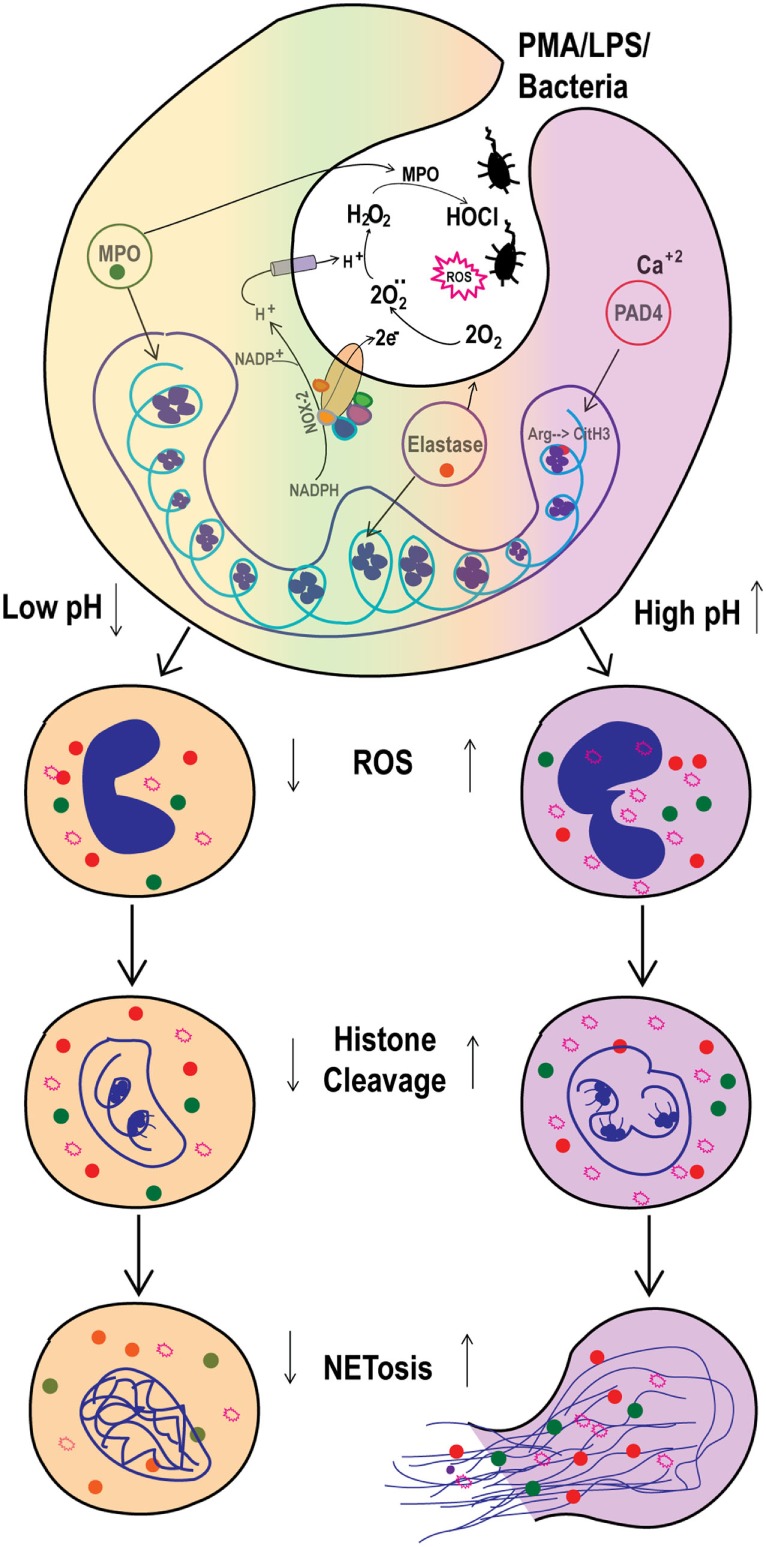
Elevated pH_i_ stimulates reactive oxygen species (ROS) production and histone H4 cleavage to regulate spontaneous and Nox-dependent NETosis. As the NADPH oxidase (Nox-2) activity triggered, electrons from cytoplasmic NADPH are translocated inside the phagosome or extracellularly, to form superoxide anions ${\text{O}}_{2}^{-\cdot }$ from O_2_ molecules. For each electron shifted into the phagosome, one proton is left in the cytoplasm, decreasing intracellular pH (pH_i_) and increasing phagosome pH. This helps in myeloperoxidase (MPO) activity to produce HOCl. Here, the increased extracellular pH alters the pH_i_ within ~10 min. The increase in pH_i_ increases ROS production and promotes histone H4 cleavage. Collectively, the increase in ROS and H4 cleavage regulate the pH-dependent spontaneous and phorbol myristate acetate (PMA)-mediated Nox-dependent NETosis.

Infection and inflammation can alter the pH of affected tissues. Open wounds, tumors, ducts from several glands, and airway secretions show variable baseline pH, which change under disease conditions (21–24). Neutrophils that extravasate into infected and/or inflamed tissues or body fluids will be exposed to various pH conditions [e.g., pH is high in pancreatitis; low in cystic fibrosis (CF) airways; and moderate in arthritis joints] (25–29). How the pH_e_ regulates Nox-dependent NETosis was not clearly established. In this study, we showed that modulation of pH_e_ led to pH_i_ adjustments and that elevating pH promoted spontaneous, as well as PMA-, LPS- and bacteria-induced, Nox-dependent NETosis. Our data provide key mechanistic details and show that high pH increased ROS production and histone cleavage to promote NETosis (Figure 9). These findings clarify the regulation of NETosis in various organs during infectious and inflammatory conditions and indicate that pH modification may alter neutrophil function in such conditions.

We and others have reported varying levels of background NETosis (4–7, 30). However, the factors and mechanisms that regulate spontaneous NETosis are not well understood. Our present study showed that pH is a key regulator of spontaneous NETosis (Figure 1). It is interesting that modulation of pH_e_ rapidly altered the pH_i_ of resting neutrophils (Figure 2). pH_e_ changed the pH_i_ at a rate of 0.05 pH unit/min, under different buffer conditions (31–34). At this rate, pH_e_ from 7.34 to 6.6 and 7.8 would equilibrate with pH_i_ within 14.8 and 9.2 min, respectively. Therefore, the values obtained in our experiments match with the rate of ~0.05 pH unit/min.

Accumulation of the product of the NADPH oxidase reaction (H^+^ ions or acidic pH) inhibits the activity of the enzyme (35, 36), and the pH optimum for Nox activity is slightly basic (pH ~7.5) (35–37). Although the amount of ROS generated at baseline is low, raising pH increased ROS in unstimulated neutrophils, albeit in smaller quantities (Figure 3). DPI is an inhibitor of Nox activity (5, 7) and suppressed spontaneous NETosis (Figure 4; Figure S2 in Supplementary Material); hence, Nox regulated the baseline NETosis. Therefore, increased baseline NETosis due to an elevation of pH was attributable to increased Nox activity and subsequent ROS production.

Citrullination of H3 can facilitate the chromatin decondensation necessary for NETosis when intracellular calcium concentrations are high (4, 5, 38). In resting neutrophils, CitH3 formation occurred only in the subpopulation of cells undergoing NETosis. This was apparent at higher pH (Figure S3 in Supplementary Material), indicating that PAD4 activity increased with increasing pH (39, 40); however, the levels of CitH3 formation was much lower in these cells compared to the conditions that increase intracellular calcium (4, 5, 7). While our manuscript is in preparation, studies showed that CitH3 increases with elevating pH in pancreas *via* increased PAD4 activity (27) and with increased bicarbonate concentrations (34). Our recent studies further showed that in response to calcium ionophores, the levels of intracellular calcium, mitochondrial ROS, CitH3 formation, and subsequent Nox-independent NETosis drastically increase with increasing pH (41). Therefore, raising pH stimulated spontaneous NETosis *via* Nox-dependent ROS production and, to a limited extent, due to the activity of citrullination of histones.

The magnitudes of the effects of pH on activated neutrophils were substantially different from resting neutrophils (Figure 1). PMA-activated neutrophils produced much higher NETosis compared to resting cells, and the effect of raised pH on NETosis was also greater. Based on morphology, MPO immunostaining and quantifying NETosis using images clearly showed typical NETosis (4, 5, 7). Therefore, pH appeared to exert different effects on PMA-mediated NETosis compared to spontaneous NETosis. SNARF fluorescence analyses showed that pH_e_ changed pH_i_ within 10 min, just as in resting neutrophils (Figure 2). However, the slope of the PMA regression line was lower than for resting neutrophils (0.093 vs. 0.124 SNARF ratio increase/pH unit). PMA-treated neutrophils are known to activate Nox, which generates H^+^ ions and reduces pH in the cytoplasm during ROS production (42, 43). Lower pH in PMA-activated compared to resting neutrophils reflected activation of Nox. Nevertheless, pH_e_ changes pH_i_ regardless of the activation status of the cells.

Reactive oxygen species levels in PMA-activated neutrophils are several orders of magnitude higher than in resting neutrophils (~3–5-fold). The pH-mediated change in ROS production was also much higher in PMA-stimulated cells than in resting neutrophils (non-linear slope with the positive factors of 268*x* + 1.7*x*^3^ for resting control vs. 616*x* + 3.9*x*^3^ for PMA stimulation; see the full equation on inset of Figure 3). These equations show that the effect of pH on ROS is higher at a high pH. DPI, a known inhibitor of Nox (5, 7), suppressed ROS production over the entire pH range tested. Therefore, Nox was the major contributor of ROS in these neutrophils (Figure 4). The effect of pH on Nox activity and ROS production reported here was consistent with previous studies on the effect of pH on ROS production (44–46) and recent reports on NETosis (13, 34), except an early study that reported the opposite effect of pH on NETosis (47). Therefore, pH was a key regulator of ROS that controls Nox-dependent NETosis.

Histone cleavage and transcription facilitate chromatin decondensation (4, 19, 48, 49). Although resting neutrophils did not show changes in histone cleavage with increasing pH, PMA-mediated NETosis showed a clear increase in histone cleavage at higher pH (Figure 5). The pH optimum of neutrophil proteases is alkaline (50, 51), and granular proteins enter the nuclei during NETosis (42). Histone H4 cleavage was reported during NETosis and is considered to be an important step involved in chromatin decondensation (19, 52). CitH3 formation is not a major event during Nox-dependent NETosis (4, 5), and immunostaining showed only a slight increase in CitH3 formation at higher pH (Figure S3 in Supplementary Material). Therefore, neutrophil proteases could effectively cleave histones at increasing pH to facilitate chromatin decondensation during agonist-induced NETosis. This is a key finding that may explain the relevance of higher pH for increasing NETosis. Although acidic pH suppresses NETosis, either sodium bicarbonate or Tris base, Tris hydroxymethyl aminomethane, is clinically referred to as THAM, effectively corrects the pH and increase NETosis (Figure 8). Correcting pH could be a promising approach for correcting the low pH-mediated suppression of NETosis.

Several studies including our own showed that LPS and bacteria can induce NETosis (4, 7, 8, 30, 53). We have recently shown that increasing LPS concentrations and bacterial load promote Nox-dependent NETosis (7). The effect of higher pH increasing NETosis is not restricted to the prototypic Nox-dependent NETosis-inducing agonist PMA. The same pH effect was seen for biologically relevant ligands such as LPS and bacteria (Figures 7 and 9). Therefore, pH was an important factor affecting NET induction by both Gram-negative (*Escherichia coli* LPS and *P. aeruginosa*) and Gram-positive (*S. aureus*) bacteria.

The findings reported in this study may suggest that regulating pH could be a therapeutic option for treating inflammatory conditions and NET-related diseases. For example, nasal and airway surface liquid in patients with CF is acidic (pH of ~5.2–7.1) compared to healthy people (pH of ~7.1–7.9) (44, 54, 55), and CF airways are often chronically infected with *P. aeruginosa* or *S. aureus* (1, 44, 56). Large numbers of neutrophils infiltrate the CF lung and accumulate in the lumen of the airways (1, 37). Although CF neutrophils undergo NETosis *ex vivo*, it remains unclear whether NETosis is compromised in CF *in vivo*. Elevating pH in CF airways could be a potential intervention aiming to improve NETosis and neutrophil homeostasis.

In summary, our present study showed that raising pH in neutrophils stimulated Nox activity and ROS production essential for NETosis, particularly during agonist-induced Nox-dependent NETosis. Neutrophil proteases have been shown to be important for NETosis (57, 58). At higher pH, proteases that entered NETotic nuclei could cleave histones more effectively due to their high pH optima. Therefore, high pH facilitates NETosis whereas low pH suppresses NETosis (Figure 9). Clinically used compounds such as sodium bicarbonate and THAM effectively raise pH and promote NETosis, suggesting the possibility of these compounds correcting defective NETosis *in vivo*.

## Ethics Statement

This study protocol for using human blood samples was approved by the ethics committee of The Hospital for Sick Children, Toronto. All the procedures including healthy human volunteer recruitment for blood donation were performed in accordance with the ethics committee guidelines. All the volunteers participating in this study gave their signed consent prior to the blood draw.

## Author Contributions

MK, LP, GC, and SV conducted experiments; MK and LP interpreted the data, prepared figures, and the manuscript; NS and HG interpreted the data and edited the manuscript. NP is the principal investigator, conceived the idea, planned experiments, supervised the study, interpreted the data, and prepared and edited the manuscript.

## Conflict of Interest Statement

NP filed a patent to regulate NETosis for treating infectious, inflammatory, and autoimmune diseases and was a consultant to Kyowa Hakko Kirin, Co., Ltd. All other authors declare that the research was conducted in the absence of any commercial or financial relationships that could be construed as a potential conflict of interest.
