# Supplementary material for: Regulating NETosis: Increasing pH Promotes NADPH Oxidase-Dependent NETosis
Source: Front Med (Lausanne). 2018 Feb 13;5:19. doi: 10.3389/fmed.2018.00019 (PMC5816902; doi:10.3389/fmed.2018.00019)
Supplement: Supplementary file 1 [file Image_1.PDF]

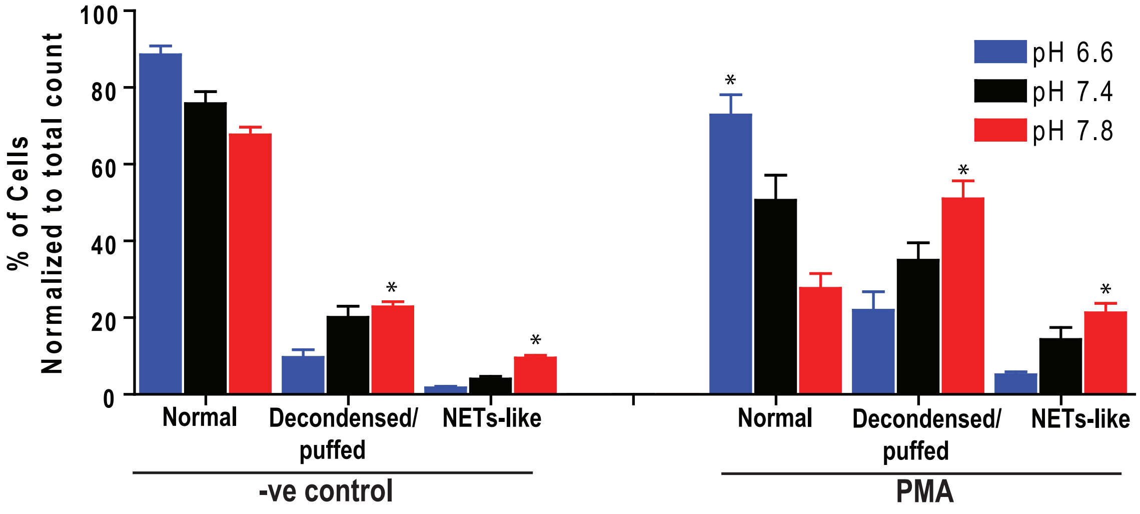

Figure S1. As shown in **Figure 1D,** the percentages of normal, decondensed or puffed and NET-like (NETotic) cells were individually calculated based on the MPO, DAPI staining, MPO-DAPI colocalization and nuclear morphology. The quantitative data of respective conditions are represented individually (n = 3; *p <0.05, comparing the condition between pH 6.6 and pH 7.8; One-way ANOVA with Tukey's multiple comparison post test).
