# Supplementary material for: Regulating NETosis: Increasing pH Promotes NADPH Oxidase-Dependent NETosis
Source: Front Med (Lausanne). 2018 Feb 13;5:19. doi: 10.3389/fmed.2018.00019 (PMC5816902; doi:10.3389/fmed.2018.00019)
Supplement: Supplementary file 2 [file Image_2.PDF]

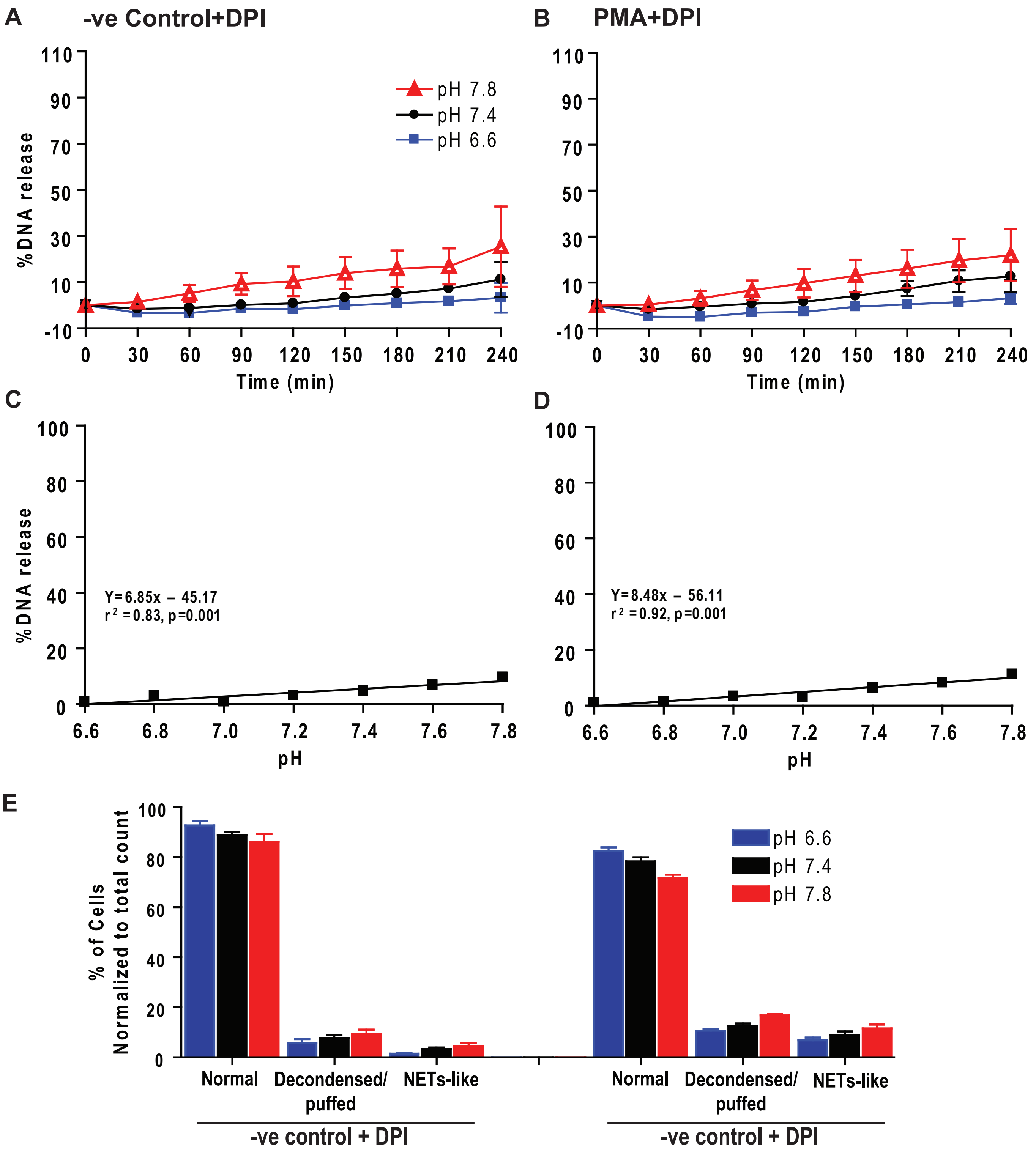

Figure S2. DPI suppressed pH-dependent increases in spontaneous and PMA-mediated NETosis. **(A-D)** Real-time tracings of NETosis kinetics and linear regression pattern showed suppression of the pH-dependent increases in spontaneous and PMA-mediated Nox-dependent NETosis. The %DNA release data of the last time point (240 min) were used in linear regression analysis (n=3-4; *, $p<0.05$; Two-way ANOVA with Bonferroni's post test; best fit linear regression analysis; p-value in each inset graph tested if the slope was different than 0; Error bars represent SEM). **(E)**, The percentages of normal, decondensed or puffed and NET-like (NETotic) cells were individually calculated and represented individually in respective conditions (n = 3; *$p<0.05$, comparing the condition between pH 6.6 and pH 7.8; One-way ANOVA with Tukey's multiple comparison post test).
