# Supplementary material for: Regulating NETosis: Increasing pH Promotes NADPH Oxidase-Dependent NETosis
Source: Front Med (Lausanne). 2018 Feb 13;5:19. doi: 10.3389/fmed.2018.00019 (PMC5816902; doi:10.3389/fmed.2018.00019)
Supplement: Supplementary file 3 [file Image_3.PDF]

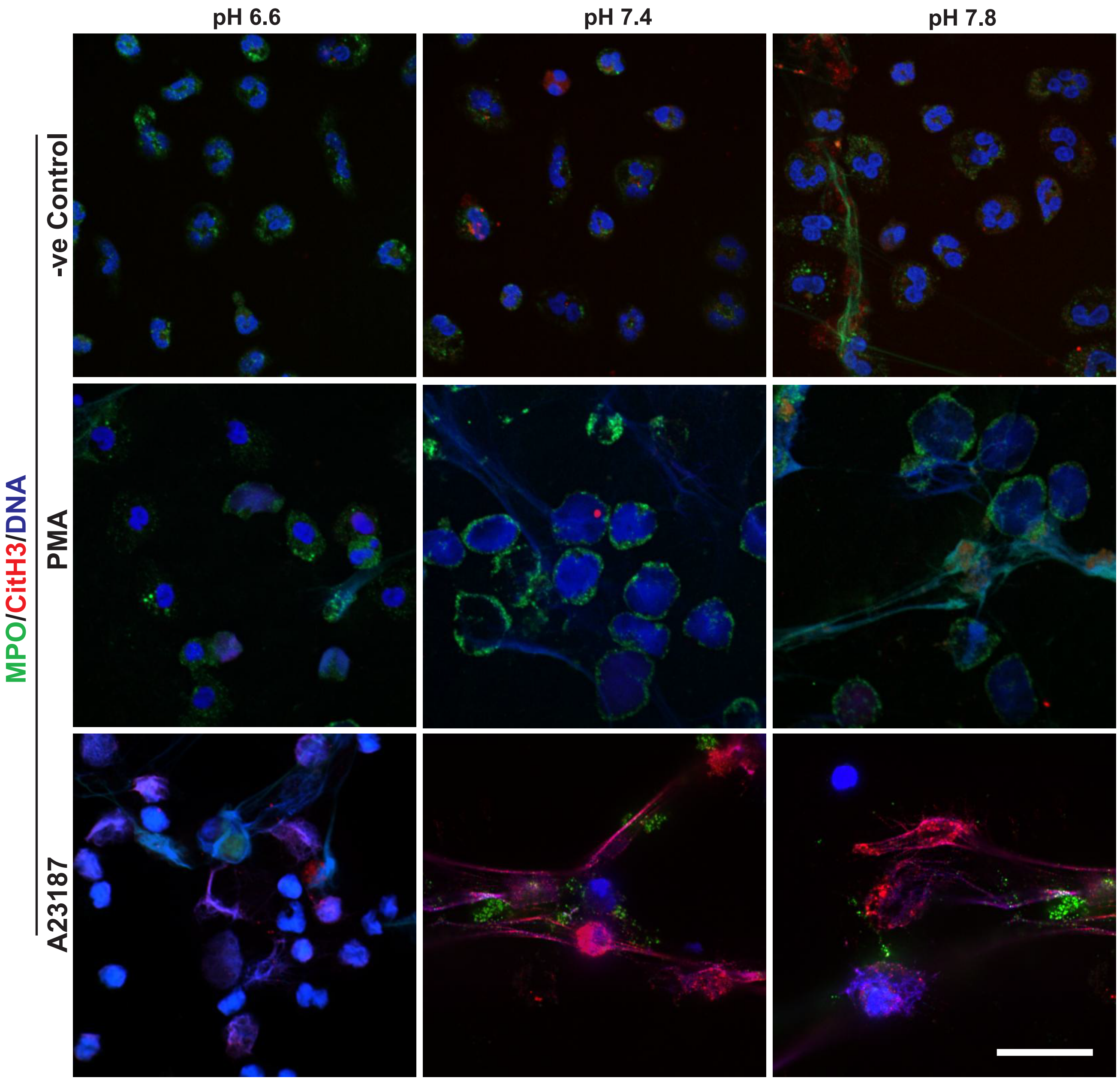

Figure S3. pH-dependent increases in spontaneous NETosis histone H3 citrullination. Immunostaining of CitH3 was performed in neutrophils activated with either media (-ve control), PMA, or A23187, resuspended in media of pH 6.6, 7.4 or 7.8. Here, A23187 is considered the positive control for histone3 citrullination. Images were analyzed and the red fluorescent intensities (for CitH3) colocalized with DNA (DAPI-blue) were considered as the degree of citrullination. The confocal images showed staining of CitH3 at higher pH conditions, particularly in spontaneous NETosis. -ve control and PMA-activated cells showed less effect on CitH3 staining at higher pH compared to the positive control. As expected, the degree of CitH3 formation was much less in Nox-dependent NETosis than calcium -ionophore mediated NETosis. The immunostaining of the neutrophils treated with calciumionophore (A23187) in different pH media were performed as part of the same experiments (Blue, DAPI staining for DNA; Red, CitH3; Green, MPO; n = 3; scale bar 20 μm).
