# Supplementary material for: Regulating NETosis: Increasing pH Promotes NADPH Oxidase-Dependent NETosis
Source: Front Med (Lausanne). 2018 Feb 13;5:19. doi: 10.3389/fmed.2018.00019 (PMC5816902; doi:10.3389/fmed.2018.00019)
Supplement: Supplementary file 4 [file Image_4.PDF]

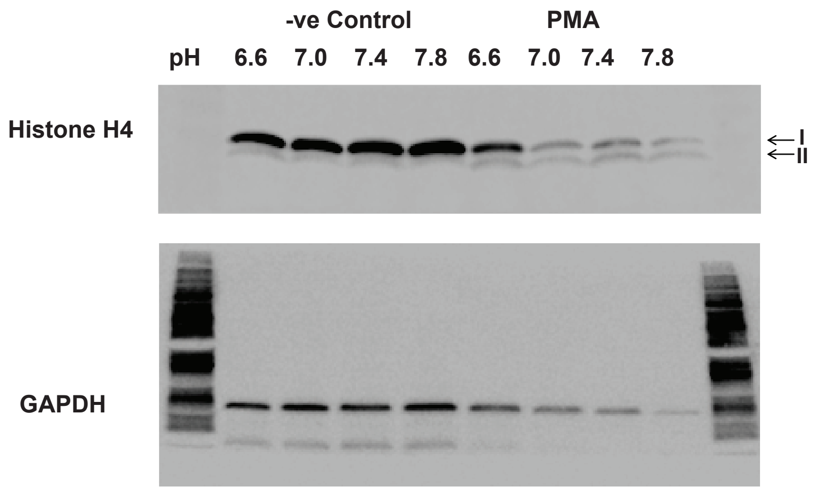

Figure S4. Elevating pH promoted Histone cleavage. Full Histone H4 immunoblot, taken from the blot sown in Figure 5.
