# Supplementary material for: Regulating NETosis: Increasing pH Promotes NADPH Oxidase-Dependent NETosis
Source: Front Med (Lausanne). 2018 Feb 13;5:19. doi: 10.3389/fmed.2018.00019 (PMC5816902; doi:10.3389/fmed.2018.00019)
Supplement: Supplementary file 5 [file Image_5.PDF]

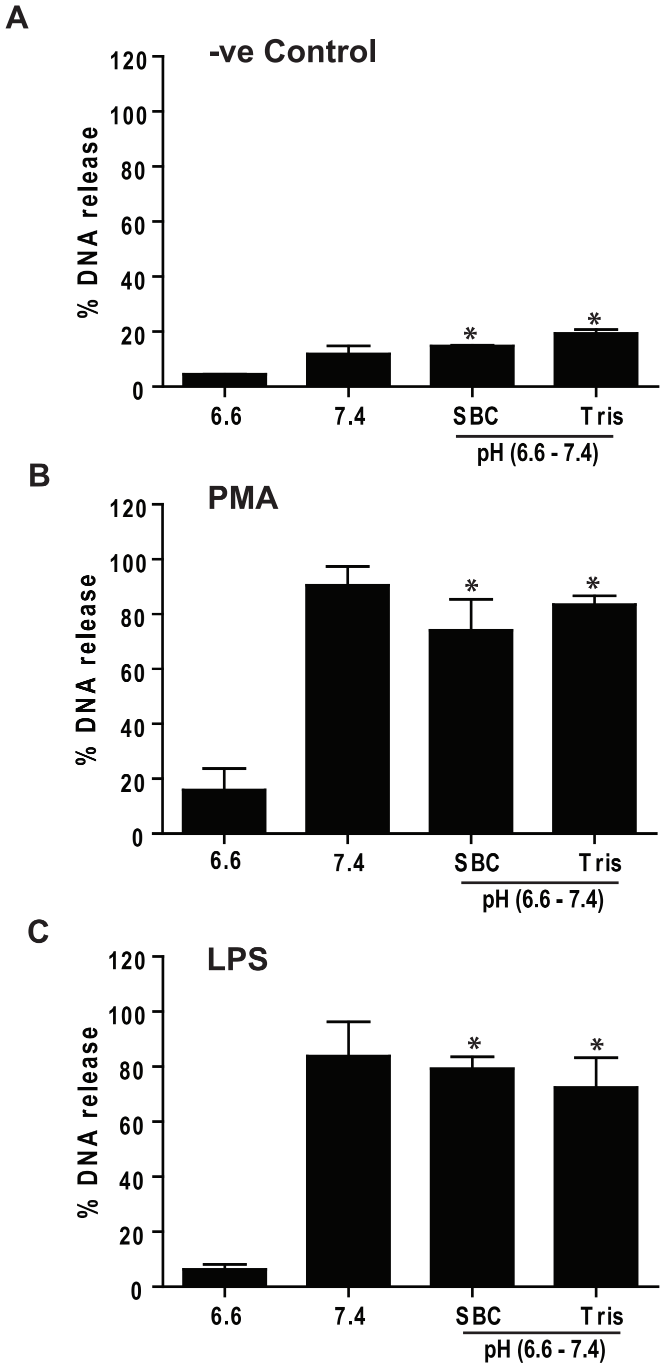

Figure S5. After pH correction either by sodium bicarbonate or Tris-base, the %DNA release bar graph at last time point (210 min) showed the significant correction in NETosis compared to pH 6.6 condition. This data is taken from the NETosis kinetics shown in Figure 8 (n=3; *, $p<0.05$; One-sample t-test).
